# The Preparation and Characterization of Chitosan/Calcium Phosphate Composite Microspheres for Biomedical Applications

**DOI:** 10.3390/polym16020167

**Published:** 2024-01-05

**Authors:** Meng-Ying Wu, Shih-Wei Huang, I-Fang Kao, Shiow-Kang Yen

**Affiliations:** 1 Department of Materials Science and Engineering, National Chung Hsing University, Taichung 402, Taiwan; slimu16882013@gmail.com (M.-Y.W.); orzroger@gmail.com (S.-W.H.);; 2 Department of Orthopedics, National Defense Medical Center, Taipei 114, Taiwan; 3 Department of Orthopedics, Taichung Armed Forces General Hospital, Taichung 404, Taiwan

**Keywords:** hydroxyapatite, chitosan, DCPD, porous microsphere, β-TCP

## Abstract

In this study, we successfully prepared porous composite microspheres composed of hydroxyapatite (HAp), di-calcium phosphate di-hydrated (DCPD), and chitosan through the hydrothermal method. The chitosan played a crucial role as a chelating agent to facilitate the growth of related calcium phosphates. The synthesized porous composite microspheres exhibit a specific surface area of 38.16 m^2^/g and a pore volume of 0.24 cm^3^/g, with the pore size ranging from 4 to 100 nm. Given the unique properties of chitosan and the exceptional porosity of these composite microspheres, they may serve as carriers for pharmaceuticals. After being annealed, the chitosan transforms into a condensed form and the DCPD transforms into Ca_2_P_2_O_7_ at 300 °C. Then, the Ca_2_P_2_O_7_ initially combines with HAp to transform into β tricalcium phosphate (β-TCP) at 500 °C where the chitosan is also completely combusted. Finally, the microspheres are composed of Ca_2_P_2_O_7_, β-TCP, and HAp, also making them suitable for applications such as injectable bone graft materials.

## 1. Introduction

Extensive research has been conducted in the field of biomaterials, encompassing various materials like metals, ceramics, polymers, composites, and the associated sciences, including chemistry, physics, polymer chemistry, polymer physics, biochemistry, and biophysics. Biopolymers, which include synthetic polymers like PLA (polylactic acid), PGA (polyglycolic acid), and PLAGA (polylactic acid–glycolic acid), as well as natural polymers including collagen, gelatin, hyaluronic acid, and chitosan, have drawn more attention recently. These materials have piqued interest due to their excellent biocompatibility and biodegradability, leading to their application in various fields, including bone tissue scaffolds [1,2,3], drug delivery systems [4,5,6,7], and artificial organ development [8,9].

**Figure 1:**
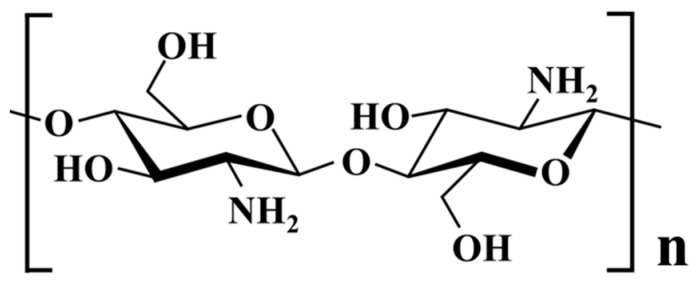
Chitosan structure.

Chitosan, as illustrated in Figure 1, is a derivative of chitin and comprises 2-amino-2-deoxy-D-glucose and 2-acetamido-2-deoxy-D-glucose units. It possesses favorable characteristics, being both biocompatible, biodegradable, and bio-adhesive [10,11,12,13]. Its utility extends to the field of bone tissue engineering, where it plays a crucial role. Chitosan can also be harnessed to create microspheres for potential drug delivery systems [14,15], employing various methods, including but not limited to spray drying [16,17,18], emulsification/solvent evaporation [19,20,21], ionotropic gelation [22,23,24], and the coacervation technique, among others [25,26].

Much like chitosan, calcium phosphate (CaP) has garnered significant attention within the realm of bone tissue engineering due to its striking resemblance to the minerals found in natural bone and its exceptional biocompatibility and bioactivity [27,28,29]. CaP encompasses a multitude of variants [30]. Hydroxyapatite (HAp, Ca_10_(PO_4_)_6_(OH)_2_), the most stable among them, serves as a primary constituent in bones and teeth. Other types of CaP include octa-calcium phosphate (OCP, Ca_8_(HPO_4_)_2_(PO_4_)_4_·5H_2_O), tetra-calcium phosphate (TTCP, Ca_4_(PO_4_)_2_O), tricalcium phosphate (TCP, Ca_3_(PO_4_)_2_), dicalcium phosphate dihydrate (DCPD, CaHPO_4_·H_2_O), and dicalcium phosphate anhydrous (DCPA, CaHPO_4_). Numerous fabrication techniques have been employed to create calcium phosphate, including hydrothermal processes [31,32,33], solid-state reactions [34,35], sol–gel synthesis [36,37], co-precipitation [38,39], and micro-emulsion synthesis [40].

For the building of bone tissue scaffolds, composite materials containing both chitosan and calcium phosphate have also been developed [41,42,43]. These composite materials have been made using a variety of manufacturing processes, such as electron electrospinning [44], ionic diffusion processes [45], freezing and lyophilization [46,47], and stepwise coprecipitation [48].

Osteomyelitis has emerged as a growing health concern, primarily attributed to bone infections, with the predominant microbial pathogen being staphylococci [49]. This condition involves inflammatory remodeling, bone destruction, and the deposition of new bone, driven by the activity of osteoblasts. Consequently, bone necrosis ensues, impacting one or more tissues and locations within the bone structure [50,51]. Osteomyelitis is categorized into three types based on its etiology. One type, resulting from local bacterial colonization, is frequently observed in post-traumatic wounds and following orthopedic surgery, especially those involving implants. The second type of osteomyelitis arises from insufficient blood supply, commonly secondary to soft tissue infection in the feet of individuals with diabetes. Meanwhile, hematogenic osteomyelitis, the third type, is more prevalent in children [52,53]. A study employing HAp as a carrier loaded with ciprofloxacin demonstrated notable antimicrobial activity, effectively enhancing the proliferation and osteogenic differentiation of rat bone marrow mesenchymal stem cells [54]. Commonly utilized antibiotics for bone infection therapy include gentamicin, vancomycin, and zyvox (linezolid) [55].

In this study, we employed the hydrothermal method to synthesize porous microspheres of CaP/chitosan in an aqueous solution containing Ca(NO_3_)_2_·4H_2_O and NH_4_H_2_PO_4_, with chitosan serving as a chelating agent. Comprehensive characterizations of these composite microspheres were conducted using techniques such as SEM, TEM, XRD, FTIR, and specific surface area porosimetry and chemisorption analysis to delve into the formation mechanism. Additionally, we examined the potential application of gentamicin, vancomycin, and zyvox-loaded microspheres in drug delivery systems.

## 2. Experimental

### 2.1. Materials

We procured Ca(NO_3_)_2_·4H_2_O (98.5%) and NH_4_H_2_PO_4_ (99.0%) from SHOWA, Tokyo, Japan, while the chitosan particles were sourced from Fluka Chemical, Buchs, Germany. To prepare a 1 wt.% chitosan (from crab shells, Sigma-Aldrich Co., St. Louis, MO, USA) solution, 3 g of chitosan was dissolved in a 297 mL aqueous solution containing 0.33% acetic acid, and the mixture was stirred for one day.

### 2.2. Synthesis of the Porous Chitosan Calcium Phosphate Microsphere (Chi-CaPM)

**Figure 2:**
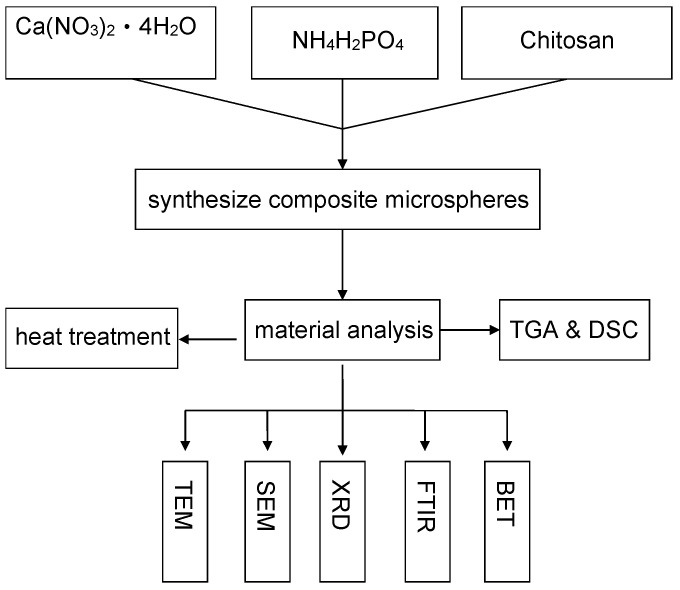
Experimental flow chart.

A composite solution was created by combining 0.042 M calcium nitrate (Ca(NO_3_)_2_·4H_2_O) and 0.025 M ammonium dihydrogen phosphate (NH_4_H_2_PO_4_), ensuring a Ca/P ratio of 1.67 for the calcium phosphate (CaP) solution. Subsequently, both the CaP solution and the 1 wt.% chitosan solution were heated to 65 °C in a water bath for an hour. They were then mixed and maintained at 65 °C for an additional half hour. During this time, white precipitates gradually settled. These precipitates were collected using a filter and subsequently subjected to 24 h of drying in an oven at 50 °C. Finally, the prepared samples underwent annealing at temperatures of 150, 300, 500, and 700 °C to investigate potential phase transformations. The experimental workflow is visually represented in Figure 2.

### 2.3. Characterization of the Powder

#### 2.3.1. Field Emission Scanning Electron Microscopy (FESEM) Analysis

The dried powders, once collected, underwent a gold coating process to facilitate observation using a FE-SEM (JSM-6700F, JEOL, Tokyo, Japan). Furthermore, the elemental composition of these powders was analyzed through the employment of an energy dispersive spectrometer (EDS, OXFORD INCA ENERGY 400, Oxfordshire, UK).

#### 2.3.2. Fourier Transform Infrared Spectroscopy (FTIR) Analysis

For the FTIR analysis, the prepared powders were mixed with KBr in a ratio of 1:100. This analysis involved examining chemical bonds within the samples, with a wave number range from 4000 to 400 cm^−1^.

#### 2.3.3. Crystal Structure Analysis

X-ray diffractions (XRD) were conducted with the Mac Science MO3x-HF Diffraction instrument from Yokohama, Japan, using Cu Kα radiation (λ = 1.5418 Å) at a voltage of 40 kV and a current of 30 mA, 2θ from 10° to 70° with a scanning rate of 2° per minute. According to Bragg’s law (2dsinθ = λ), there could be diffraction patterns. Subsequently, these diffraction patterns were analyzed and cross-referenced with the International Centre for Diffraction Data (ICDD) (Newtown Square, PA, USA) to facilitate the identification of the crystal structure.

#### 2.3.4. Brunauer–Emmett–Teller (BET) and Barret–Joyner–Halender (BJH) Analysis

The specific surface area of the samples was assessed through nitrogen adsorption using a Micromeritics ASAP 2010 nitrogen adsorption apparatus. Employing the multipoint BET method, the specific surface area was calculated by analyzing adsorption data within the relative pressure (P/P0) range of 0.02 to 0.45. The BJH method was applied to establish the pore size distribution from the desorption isotherm. The average pore size and pore volume were calculated using the nitrogen adsorption volume at a relative pressure (P/P0) of 0.972.

#### 2.3.5. Transmission Electron Microscopy (TEM) Analysis

TEM images and selected area diffraction (SAD) patterns were acquired using a JEOL JEM-200CX TEM (Tokyo, Japan) with an acceleration voltage of 160 kV. To prepare the samples for analysis, the solution precipitates were cast onto copper grids with a carbon coating, left to dry naturally, and subsequently affixed to the microscope’s sample holder.

#### 2.3.6. Inductive Coupled Plasma–Mass Spectrometry (ICP-MS) Analysis

To adhere to American Society for Testing and Materials (ASTM) standard F1185-03 [56], the powder underwent analysis using ICP-MS. Specifically, 0.5 mg of Chi-CaPM was dissolved in 15 mL of 5% HCl solution. This ICP-MS analysis served to determine the Ca/P ratio and assess the concentration of potentially harmful metal ions, including Pb, Cd, Hg, and As, present within the prepared sample.

### 2.4. Thermogravimetric Analysis (TGA)

TGA was executed with a heating rate of 10 °C/min under both air and N_2_ atmospheres, reaching temperatures up to 800 °C. The weight variation with temperature was recorded, revealing the weight loss during heating. Additionally, we performed differential scanning calorimetry (DSC) under the same heating conditions in air and N_2_ atmospheres, extending up to 800 °C. This analysis enabled us to reveal endothermic and exothermic reactions and estimate the enthalpy.

## 3. Results and Discussion

### 3.1. Crystal Structure and Phase Transformation

**Figure 3:**
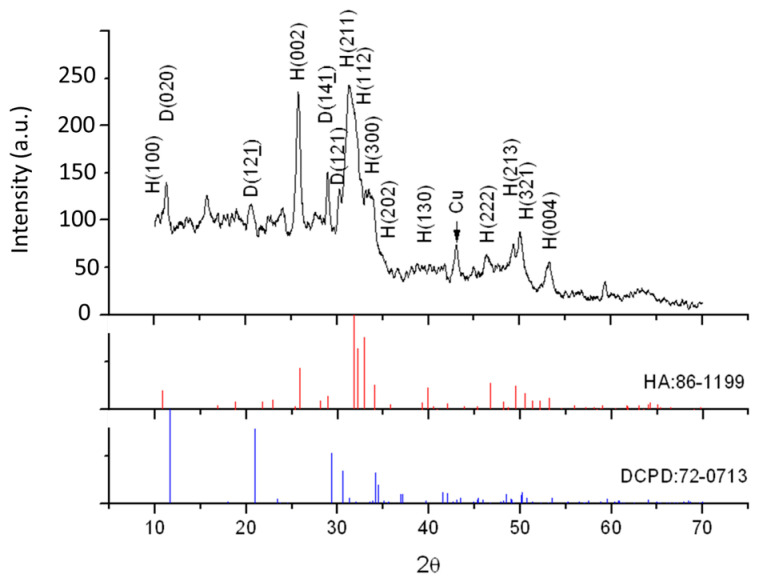
X-ray diffraction patterns of the as-prepared Chi-CaPM.

The XRD diagram of Chi-CaPM is shown in Figure 3 and was also compared with the ICDD card No. 86-1199 and No. 72-0713 peaks. The diffraction peaks of crystal planes were 10.842° (100), 25.883° (002), 31.792° (211), 32.206° (112), 32.929° (202), 46.726° (222), 49.508° (213), 50.523° (321), and 53.220° (004) of hydroxyapatite. Also, the diffraction peaks at 11.65° (020), 20.948° (121), 29.296° (141), and 30.543° (121) of DCPD were detected. The higher background intensity from 10° to 30° is a result of the amorphous nature of chitosan. 

**Figure 4:**
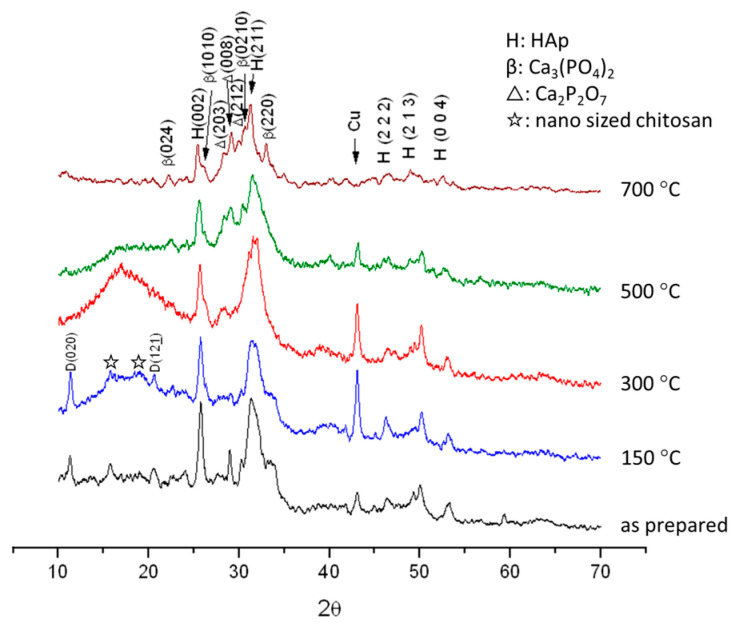
XRD diagrams of the heat-treated Chi-CaPM.

The XRD pattern of specimens annealed at 150, 300, 500, and 700 °C is shown in Figure 4. When heated at 150 °C, there are two weak peaks and one broad peak from 15° to 20°, where the peaks at 16° and 19° represent nano-sized chitosan [57]. When heated at 300 °C, the intensity of the broad peak becomes stronger, due to amorphous structures caused by the condensation of OH and/or NH bonds. Moreover, no post-annealed sample exhibited a broad peak; it was weaker than the heat-treated sample. There is no broad peak from 15° to 20° at 500 °C and 700 °C because of the complete oxidation of the dehydrated and/or condensed chitosan. The DCPD peak vanishes, and Ca_2_P_2_O_7_ (pyrophosphate) appears at 300 °C, signifying the transformation of DCPD into DCPA and initiating the transformation into Ca_2_P_2_O_7_, as depicted in reactions (1) and (2). At 500 °C, reaction (3) initiates to form β-TCP. Finally, the crystallinity of β-TCP is further enhanced at 700 °C:

CaHPO_4_·2H_2_O → CaHPO_4_ + 2H_2_O(↑) (1)

2CaHPO_4_ → Ca_2_P_2_O_7_ + H_2_O(↑) (2)

Ca_2_P_2_O_7_ + Ca_10_(PO_4_)_6_(OH)_2_ → 4Ca_3_(PO_4_)_2_ + H_2_O(↑) (3)

**Figure 5:**
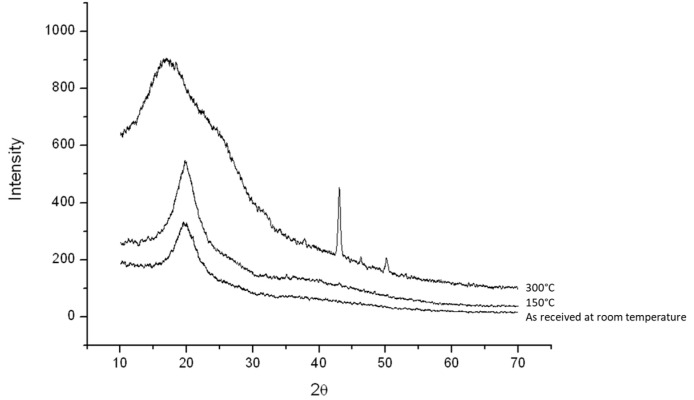
XRD diagrams of the heat-treated chitosan.

The detailed transformation of pure chitosan from room temperature to 300 °C is shown in Figure 5, indicating the type Ⅱ crystal structure of the as-received chitosan which was annealed at 150 °C, while it becomes a completely amorphous structure at 300 °C like that found in Figure 4 (the red one).

**Figure 6:**
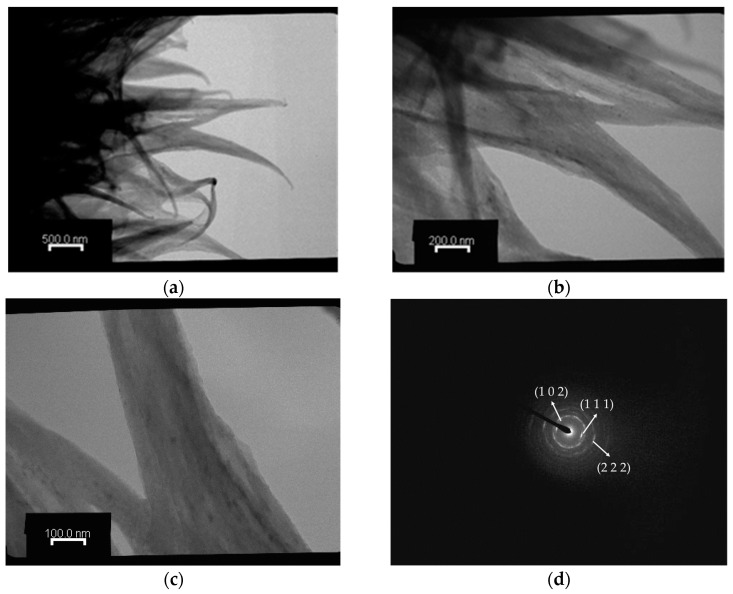
TEM image of the Chi-CaPM at (**a**) 20,000× magnification (**b**) 50,000× magnification, and (**c**) 100,000× magnification and (**d**) the SADP of the Chi-CaPM.

The TEM observations of the Chi-CaPM and select area diffraction pattern (SADP) are shown in Figure 6. From the TEM image, it can be seen that the edge of Chi-CaPM looks like grass, and the grass is composed of several nano-sticks. The select area diffraction pattern (SADP) confirms that Chi-CaPM reveals a ring-shaped diffraction pattern from (102) planes of hydroxyapatite. Additionally, there are two pairs of arc-shaped diffraction patterns from (111) and (222). This phenomenon indicates that hydroxyapatite has a high preferential orientation. In other words, hydroxyapatite precipitates on chitosan with a (111) texture parallel to the long axis of chitosan.

**Figure 7:**
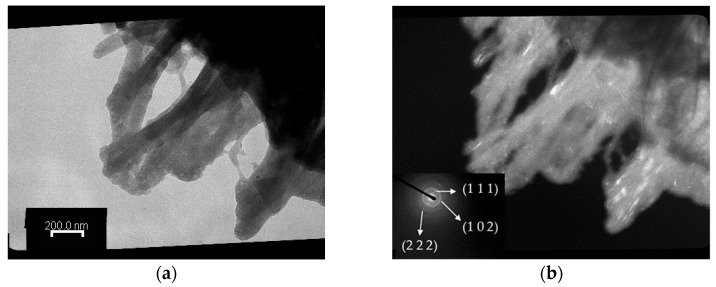
TEM images of the Chi-CaPM treated at 500 °C at 50,000× magnification (**a**) in bright field and (**b**) dark field conditions including the SADP.

The TEM observations of Chi-CaPM after annealing at 500 °C for one hour are shown in Figure 7. The grass-like edge transforms into a rod shape. Like Figure 6d, its SADP exhibits a ring-shaped diffraction pattern from the (102) planes of hydroxyapatite and two pairs of arc-shaped diffraction patterns from the (111) and (222) planes of hydroxyapatite.

**Figure 8:**
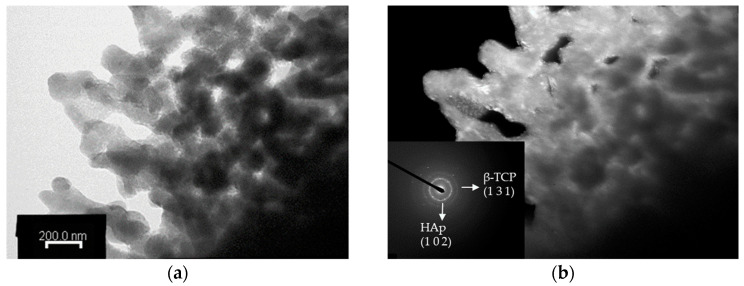
TEM images of the Chi-CaPM treated at 700 °C at 50,000× magnification (**a**) in bright field and (**b**) dark field conditions including the SADP.

The TEM observation of porous Chi-CaPM after annealing at 700 °C for one hour is shown in Figure 8. The cylinders transform into round particles. The ring-shaped diffraction pattern is still observed from the (102) plane of hydroxyapatite, as shown in Figure 6d. However, the arc-shaped diffraction patterns disappear, and a few bright points are found. This indicates the grain growth of hydroxyapatite, and some diffraction rings of β-TCP such as the (131) plane are also observed, clearly revealing the phase transformation from hydroxyapatite and Ca_2_P_2_O_7_ to β-TCP in reaction (3).

**Figure 9:**
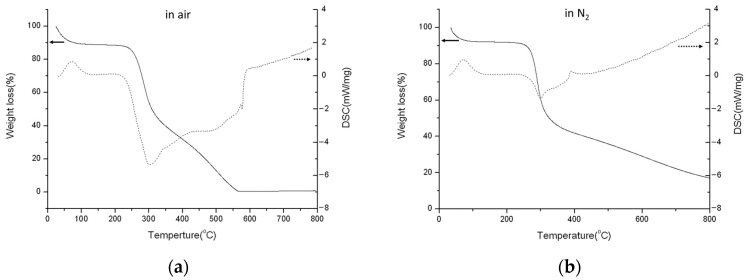
TGA (solid line)/DSC (dotted line) profiles of chitosan in (**a**) air and (**b**) N_2_.

The TGA/DSC diagrams of chitosan carried out in air or N_2_ are shown in Figure 9a,b. From Figure 9a, they reveal three steps of weight loss. The first step, accounting for about 12% of the loss, is the evaporation of H_2_O, corresponding to the endothermic peak below 100 °C in the DSC diagram. The second step, involving a loss of about 42% at around 300 °C, results from the dehydration and/or condensation of chitosan, and this corresponds to the exothermic peak at around 300 °C. The third step is the further oxidation of the dehydrated and/or condensed chitosan in the air, corresponding to the exothermic region from 350 °C to 600 °C in the DSC diagram. These results are completely consistent with those discussed in the XRD as shown in Figure 3, Figure 4 and Figure 5. It is evident that the weight loss rate above 300 °C in N_2_ is slower than in air, primarily due to less oxidation in N_2_ as shown in Figure 9b.

**Figure 10:**
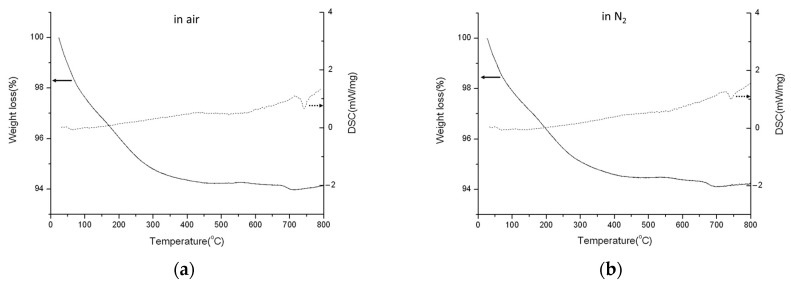
TGA (solid line)/DSC (dotted line) profiles of HAp in (**a**) air and (**b**) N_2_.

The TGA/DSC diagrams of HAp are shown in Figure 10a,b. The major weight loss is only 6% from ambient temperature to 300 °C, resulting from the evaporation of H_2_O adsorbed in HAp.

In this temperature range, there are no obvious endothermic or exothermic peaks until the temperature up to 700 °C in the DSC diagram. The exothermic peak around 700 °C corresponds to reaction (4). Additionally, a slight weight loss is observed in the TGA diagram, ascribed to the dehydration in reaction (4). There is no noticeable difference between the diagrams in air and in N_2_ because there is no oxidation occurring for HAp.

Ca_10_(PO_4_)_6_(OH)_2_ → 3Ca_3_(PO_4_)_2_ + CaO+ H_2_O (↑) (4)

**Figure 11:**
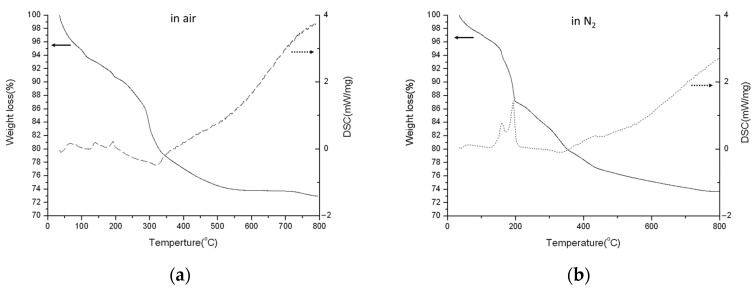
TGA (solid line)/DSC (dotted line) profiles of Chi-CaPM in (**a**) air and (**b**) N_2_.

The TGA/DSC diagrams of the prepared Chi-CaPM from room temperature to 800 °C in air and N_2_ are shown in Figure 11a,b. There are three endothermic peaks below 250 °C, attributed to the evaporation of adsorbed water, the dehydration from DCPD into DCPA around 140 °C, and the further transformation of DCPA into Ca_2_P_2_O_7_ around 190 °C, the latter two corresponding to reactions (1) and (2), respectively, which are not found in the TGA/DSC diagrams of HAp. Consistently, the XRD peaks of the DCPD are found in the as-prepared and annealed samples at 150 °C while they gradually disappear at 300 °C, as shown in Figure 4 where Ca_2_P_2_O_7_ is found.

### 3.2. Chemical Bondings and Compositions

**Figure 12:**
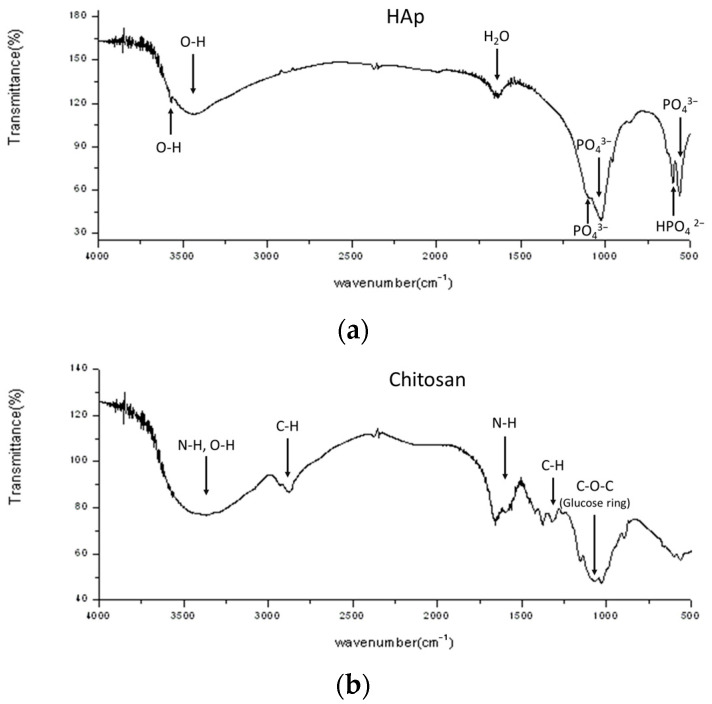

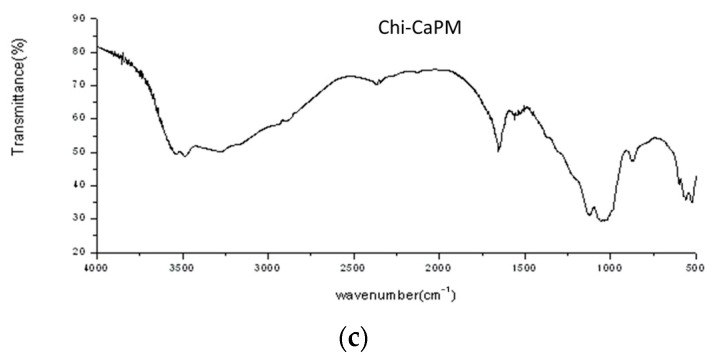
The FTIR spectra of (**a**) HAp, (**b**) chitosan, and (**c**) Chi-CaPM.

The FTIR spectra of HAp, chitosan, and the prepared Chi-CaPM microsphere are shown in Figure 12a–c. In the spectrum of HAp shown in Figure 12a, OH groups are found at wavenumbers in the range of 3600 to 3150 cm^−1^ and an explicit peak at 3570 cm^−1^. The wavenumber at 1650 cm^−1^ corresponds to H_2_O bending, which is different from the peak of CO_3_^2−^ (1460–1530 cm^−1^) [58]. Phosphate absorption bands at 1092, 1033, 964, 606, and 564 cm^−1^ are characteristics of a typical FTIR spectrum for HAp. The wavenumbers at 3300~3500 cm^−1^ (N-H and/or O-H), 2800~2900 cm^−1^ (C-H), 1590 cm^−1^ (N-H), 1382~1413 cm^−1^ (C-H), and 1160~1040 cm^−1^ (C-O-C, glucose ring) corresponding to the characteristics of chitosan are shown in Figure 12b. It was found that the chemical bonding of the prepared Chi-CaPM, as shown in Figure 12c, is a combination of HAp and chitosan, revealing both the absorption peaks of HAp and chitosan.

**Figure 13:**
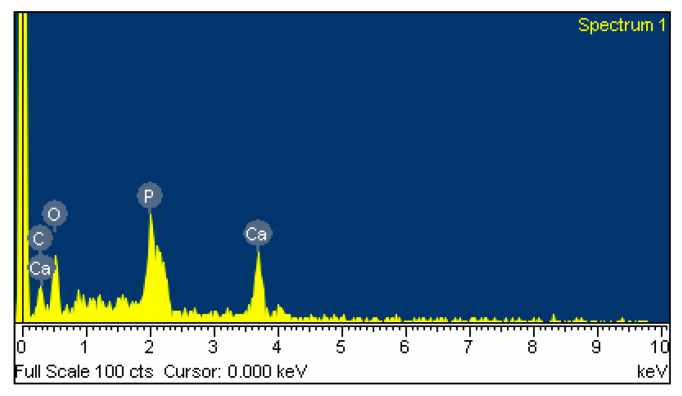
EDS spectra of Chi-CaPM.

The EDS spectra indicate that the microsphere is mainly composed of C, O, P, and Ca elements, as shown in Figure 13. Their detailed contents are listed in Table 1. The ratio of Ca to P is 1.43. This means that the calcium phosphate in the composite microsphere is composed of 64.5% HAp and 35.5% DCPD. Combining TGA/DSC in Figure 9 and Figure 11, the composite sphere is entirely composed of 16% chitosan, 29.8% DCPD, and 54.2%.

The ICP-MS of the prepared microspheres is shown in Table 2, indicating the elements contained in (a) original 5% HCl, (b) Chi-CaPM dissolved in 5% HCl, and (c) derived from (b)–(a) for pure Chi-CaPM. According to the ASTM standard [56], the maximum allowable limit for all heavy metals is 50 ppm. The prepared porous Chi-CaPM in this study is well below this limit. Additionally, the Ca/P ratio was determined to be 1.47, which is approximate to that of the EDS analysis.

### 3.3. Surface Morphology, Specific Surface Area, and Pore Volume

**Figure 14:**
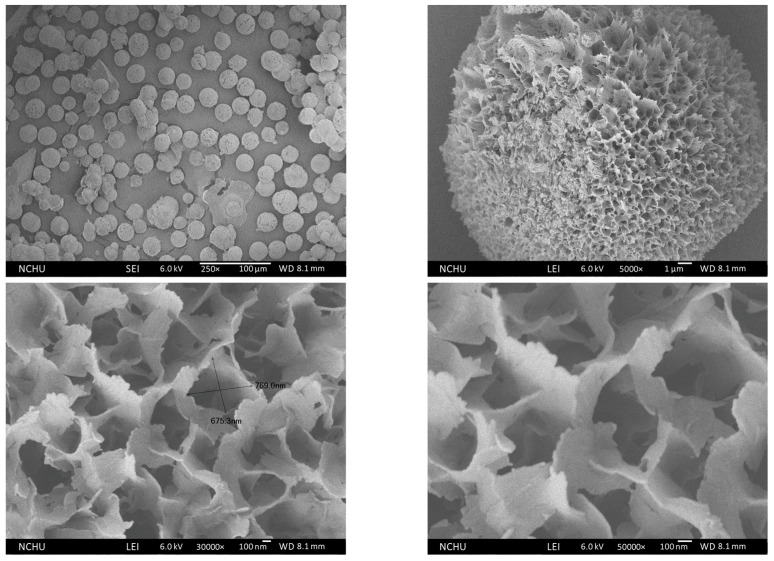
Morphology of Chi-CaPM.

The SEM observation of Chi-CaPM is shown in Figure 14. It was found that Chi-CaPM is full of pores assembled by petal-like flakes. The average granularity of porous Chi-CaPM is about 25 μm, as shown in the upper two, and the pore size on the surface is about 0.7 μm, as shown in the lower two. From the higher magnification TEM image, the petal-like flakes are composed of several nano-sized rods, as shown in Figure 6. 

**Figure 15:**
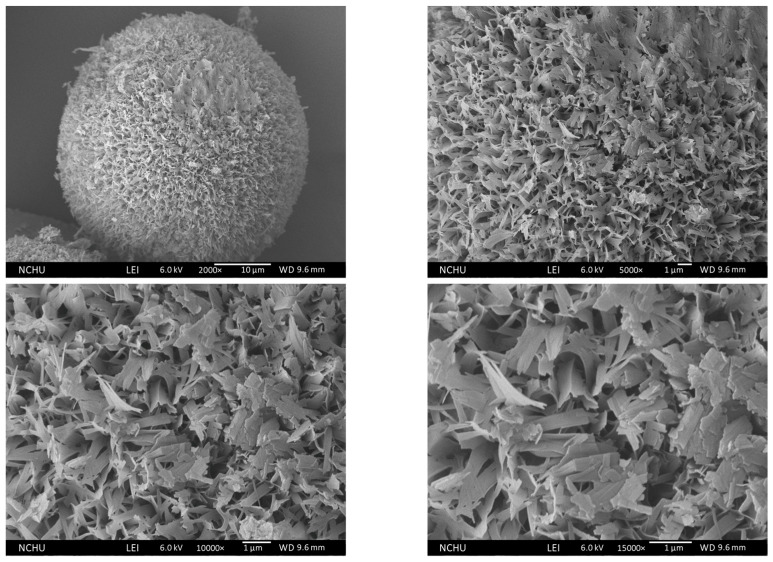
Morphology of 150 °C Chi-CaPM.

After annealing at 150 °C for one hour, as shown in Figure 15, the submicron-sized pores collapse, and the petal-like flakes transform into dispersive narrow plates.

**Figure 16:**
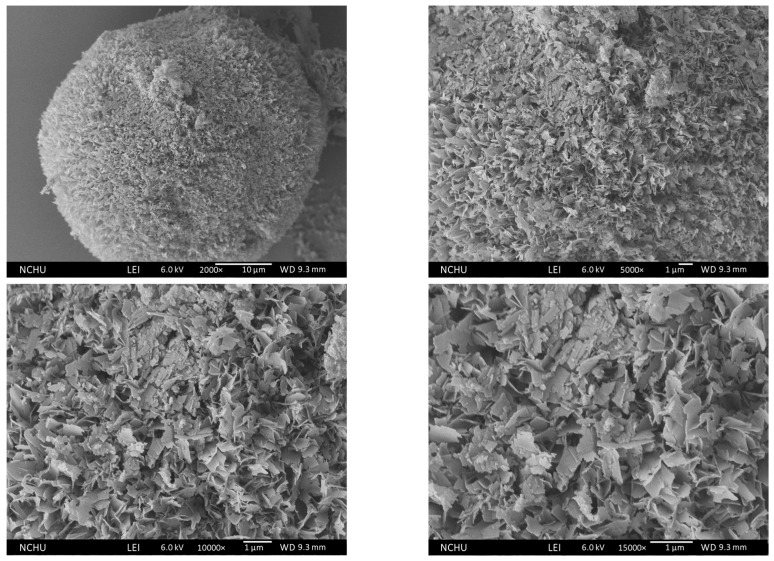
Morphology of 300 °C Chi-CaPM.

Similar results were found at 300 °C, as shown in Figure 16. The flower-like flakes collapsed into smaller pieces due to the decomposition of chitosan.

**Figure 17:**
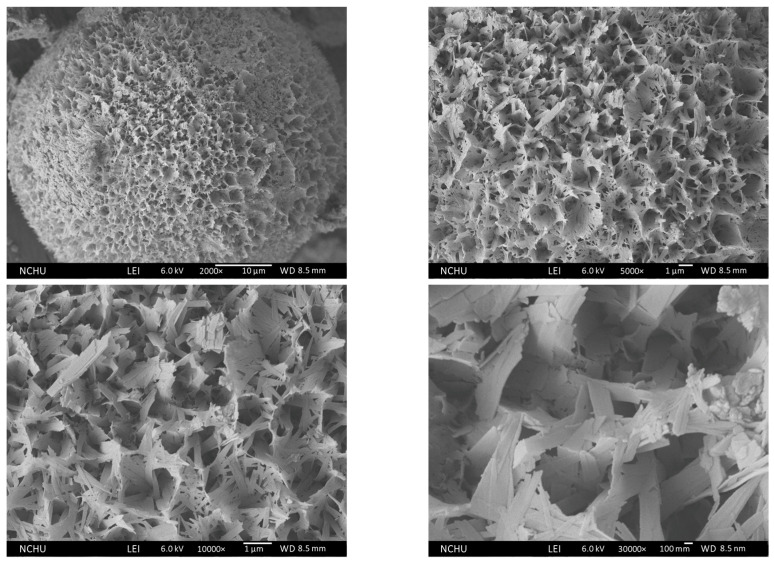
Morphology of 500 °C CaPM.

The coalescence of dispersive narrow plates into wider ones was found at 500 °C for one hour, and the collapsed pieces coalesced into larger petal-like flakes again, as shown in Figure 17.

**Figure 18:**
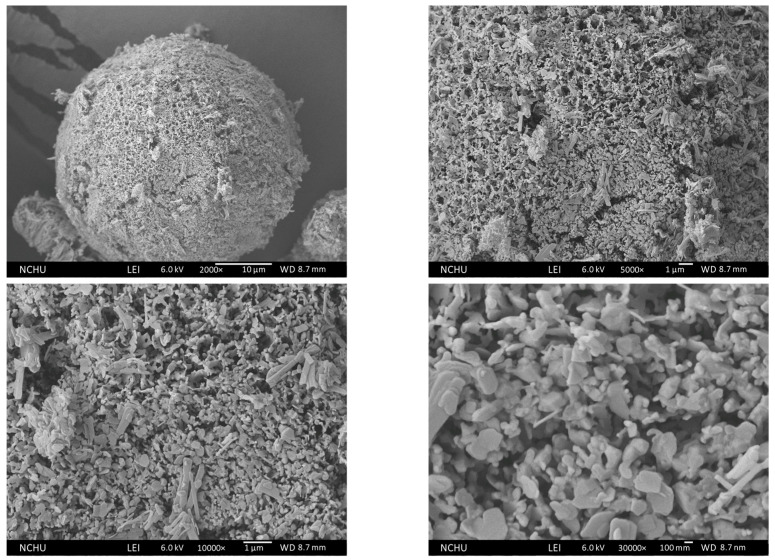
Morphology of 700 °C CaPM.

After annealing at 700 °C for one hour, the surface morphology of the microsphere changed significantly. The plates transformed into round particles, as shown in Figure 18. Obviously, the variations in the surface morphology of the composite microsphere were all consistent with the phase transformation of calcium phosphate and the decomposition of chitosan discussed in Section 3.1.

**Figure 19:**
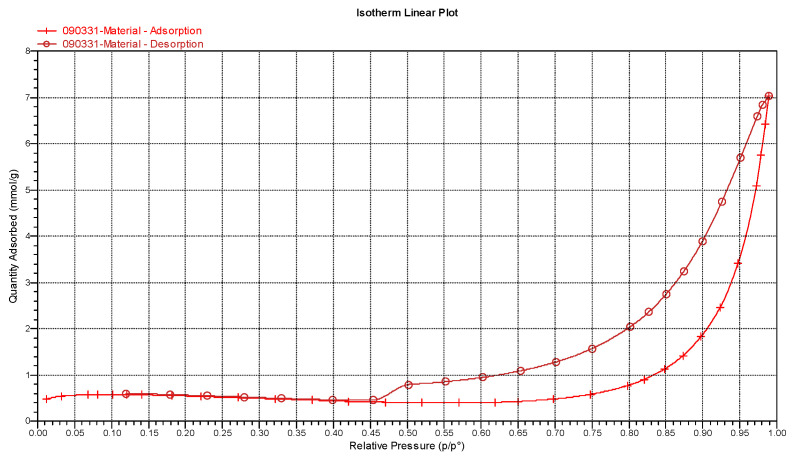
BET nitrogen adsorption/desorption isotherm.

The BET nitrogen adsorption/desorption isotherm is shown in Figure 19. The typical hysteresis loop is usually found in multi-layer adsorption. Four characteristic adsorption curves are illustrated in a prior report [59]. The H1 type results from clustered invariant spheres constituting material with porosity. H2 is not defined clearly now, as various factors could be considered. H3 is the result of porosity assembled by micro flakes, and the H4 hysteresis loop is created by a narrow aperture [59]. The hysteresis loop of the microspheres in this study reveals the BET nitrogen adsorption/desorption isotherm, like H3, as shown in Figure 19.

**Figure 20:**
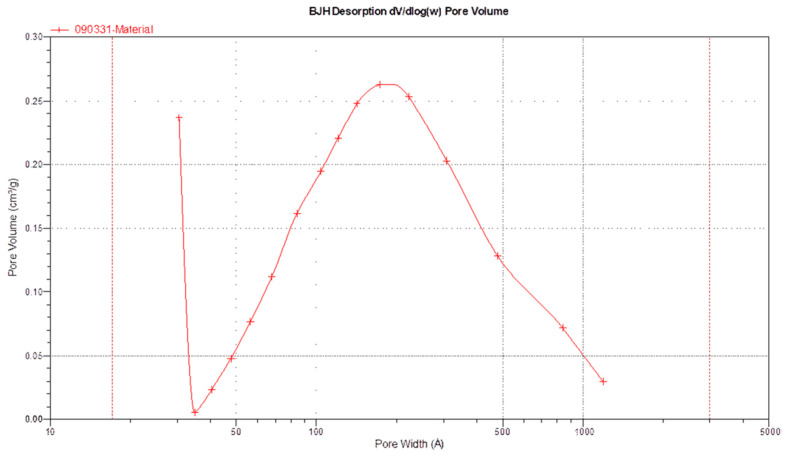
BJH desorption pore.

According to the BET theory analysis, as listed in Table 3, the surface area value is 38.16 m^2^/g. There are also studies that synthesize spherical HAp using the co-precipitation method, the fast precipitation method, and the hydrothermal method—the same as we used. The HAps they synthesized can achieve surface areas of 126 m^2^/g [60], 164.73 m^2^/g [61], and 105.21 m^2^/g [62], with corresponding average particle sizes of 1.7 μm, 1.5 μm, and 3 μm, respectively. In comparison, the average size of Chi-CaPM is 30 μm. The surface areas of their synthesized HAp are all higher than that of Chi-CaPM, with the particle size of 30 μm in this study. As the particle size decreases, the surface area increases. From Figure 20, the pore size analysis by BJH desorption varies from 3 to 100 nm. Two peaks of volume distribution are found at 3 and 20 nm, indicating significant porosity and a high surface area, which makes it a strong potential drug carrier. When using the BJH method to measure the surface area of pores ranging from 1.7 to 300 nm in width, the specific surface area values are 33.32 m^2^/g and 76.29 m^2^/g for adsorption and desorption, respectively. The pore volume is 0.24 cm^3^/g for adsorption and 0.24 cm^3^/g for desorption.

### 3.4. Antibiotic Loading and Release

**Figure 21:**
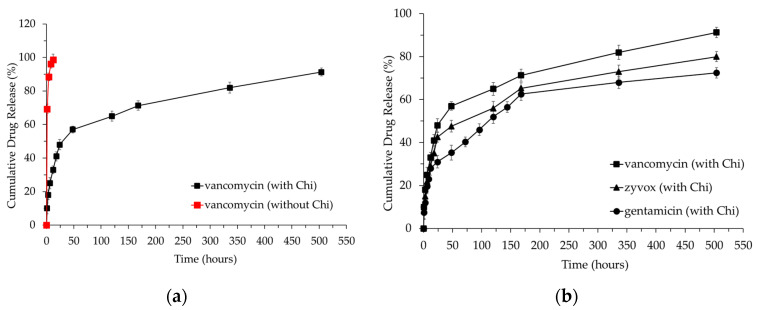
The cumulative drug release of (**a**) V-Chi-CaPM with (black one) and without (red one) additional chitosan and (**b**) V-Chi-CaPM, Z-Chi-CaPM, and G-Chi-CaPM with additional chitosan.

To investigate whether the addition of chitosan affects drug release, we first immersed Chi-CaPM in a drug solution without additional chitosan and mixed it at 37 °C for one day. The drug-to-Chi-CaPM weight ratio is 1/3, and the resulting mixture was then dried in a 37 °C oven. The second experiment group involved adding an additional 20 mg of chitosan to the vancomycin-Chi-CaPM (V-Chi-CaPM) after 12 h of mixing. The blend was further mixed for an additional 12 h before being dried. Afterwards, the loading method with the better release profile was chosen for the encapsulation of zyvox (Z-Chi-CaPM) and gentamicin (G-Chi-CaPM). The drug-loaded powders were placed in a 100 mL phosphate-buffered saline (PBS) solution at 37 °C with an 80 rpm shaking rate. At predefined time intervals, 1 ml of the medium was removed and an equal volume of fresh PBS buffer was put in. The collected medium concentration was measured using a UV/VIS spectrometer (U-3010, HITACHI, Tokyo, Japan). The in vitro cumulative drug release curves are shown in Figure 21. The detailed cumulative calculation is described in a previous report [63].

The results in Figure 21a indicate that in the absence of chitosan, the drug is completely released within 12 h. However, with the addition of chitosan, the release period of the drug extends to up to 21 days. This prolonged release time is attributed to the hydrogen bonding interactions between chitosan and vancomycin, as well as those between chitosan and HAp. These hydrogen bonding interactions enhance the connection between the drug and the carrier, forming a more stable composite structure that consequently extends the drug release time [64,65,66,67]. This outcome demonstrates the significant impact of adding chitosan on regulating drug release, leading to the incorporation of chitosan into the subsequent release experiments for the three drugs.

After 21 days of experimentation, the accumulative release amounts of vancomycin, zyvox, and gentamicin reached 91%, 80%, and 72% as shown in Figure 21b. In general, in vitro drug release exhibits a three-phase profile, including the first phase occurring within 24 h, which represents the initial drug burst and the fastest release rate, the second phase lasting from days two to seven involving the steady release rate, and the third phase occurring after week one and then displaying the slowest release rate. The disparities in release quantities can be attributed to the zeta potential of chitosan and each drug. Both chitosan and vancomycin have positive zeta potentials, while zyvox and gentamicin have negative zeta potentials [68,69,70,71]. The repulsion between the positive zeta potentials of chitosan and vancomycin leads to the fastest release of vancomycin. Additionally, gentamicin has a slightly more negative zeta potential than zyvox, resulting in stronger attraction via Coulombic force between gentamicin and chitosan compared to zyvox and chitosan. Consequently, the release of gentamicin is relatively slower. Basically, these Chi-CaPMs have revealed their potential as drug carriers since the characteristics of Chi-CaPMs include a great pore volume and a high specific surface area.

## 4. Conclusions

The porous Chi-CaPM was successfully prepared using the hydrothermal method. Characterization through XRD, FTIR, ICP-MS, and TGA/DSC identified that the porous calcium phosphate/chitosan composite microsphere consists of 54.2 wt.% HAp, 29.8 wt.% DCPD, and 16 wt.% chitosan. The chitosan transforms into the dehydrated and/or condensed one and the DCPD transforms into Ca_2_P_2_O_7_ at 300 °C. Then, Ca_2_P_2_O_7_ initially combines with HAp to transform into β-TCP at 500 °C where the chitosan is also completely combusted. Finally, the microspheres are composed of Ca_2_P_2_O_7_, β-TCP, and HAp, making them suitable for applications such as injectable bone graft materials. 

TEM observations and SADP analyses illustrate that the grass-like edge of the porous calcium phosphate/chitosan composite microsphere consists of HAp, which precipitated along chitosan with a (111) texture. The FTIR results of the prepared microsphere reveal both chitosan and CaP chemical bonds. The porous calcium phosphate/chitosan composite microsphere exhibits a high specific surface area of 38.16 m^2^/g and a significant pore volume of 0.244 cm^3^/g.

All of the in vitro drug releases of gentamicin, vancomycin, and zyvox follow three-phase profiles, including the initial drug burst within 24 h, the second phase with a steady drug release rate from days two to seven, and the third phase consisting of a lower steady release rate from week two to week three. With their great pore volume and high specific surface area, the prepared microspheres demonstrate strong potential for use as drug carriers.

## Figures and Tables

**Table 1:** The compositions of Chi-CaPM derived from the EDS analysis.

Element	wt.%	at.%
C	15.28	25.17
O	41.52	51.33
P	15.11	9.64
Ca	28.09	13.86
Totals	100.00	100.00

**Table 2:** ICP-MS of the element analysis.

	(a) 5% HCl	(b) Chi-CaPM Dissolved in 5% HCl	(c) Pure Chi-CaPM
P	0.0295	109	108.97
Ca	0.015	160	159.99
As	0.0773	0.1553	0.0780
Cd	0.00093	0.01233	0.0114
Hg	0.00126	0.02308	0.0218
Pb	0.0257	0.1451	0.1194

Unit: ppm (part per million).

**Table 3:** BET analysis of surface area, pore volume, and pore size.

	BET Adsorption Theory	BJH	
		Adsorption	Desorption
Surface area	38.16 m^2^/g	33.32 m^2^/g	76.29 m^2^/g
Pore volume		0.244 cm^3^/g	0.244 cm^3^/g
Pore size	18.49 nm	29.29 nm	12.80 nm

## Data Availability

Data are contained within the article.
